# Adult-onset transient pseudohypoaldosteronism secondary to obstructive nephropathy: a case report

**DOI:** 10.1007/s11255-024-03975-0

**Published:** 2024-02-20

**Authors:** Mari Yamamoto, Fumitoshi Sakamoto, Hiroki Ikai, Yoshiro Fujita

**Affiliations:** 1 Department of Rheumatology and Nephrology, Chubu Rosai Hospital, 1-10-6 Komei, Minato-ku, Nagoya, Aichi, 455-8530 Japan; 2 Department of Urology, Chubu Rosai Hospital, 1-10-6 Komei, Minato-ku, Nagoya, 455-8530 Japan

Editor,

Pseudohypoaldosteronism (PHA) and secondary (transient) PHA (TPHA) are considered etiologies of type 4 renal tubular acidosis (RTA), because they are associated with hyperkalemia and metabolic acidosis [1]. Aldosterone resistant-TPHA and hypoaldosteronism-induced type 4 RTA have distinct pathogenic mechanisms. A rare case of ureteral cancer-induced hydronephrosis leading to adult-onset TPHA is presented, the first to demonstrate hormone-level alterations pre- and post-ureteral obstruction.

A 70-year-old man, with 60 pack-year smoking history, chronic obstructive pulmonary disease, and suspected rheumatoid arthritis, taking iguratimod (50 mg/day), presented with fatigue and hematuria persisting for 2 months. Antibiotics administration for suspected urinary tract infection showed no improvement. As his renal function worsened (creatinine, 0.6–1.8 mg/dL), he was hospitalized.

On admission (day 0), blood and urine examinations revealed renal dysfunction, with hematuria, proteinuria, normal anion gap (AG) metabolic acidosis, hyponatremia, and hyperkalemia. Computed tomography revealed bilateral ureteral dilatation and hydronephrosis, with no obvious obstruction. Normal AG metabolic acidosis with hyperkalemia indicated type 4 RTA. However, high renin (70.5 [normal, 2.5–21.4] ng/mL/h) and aldosterone (678.2 [normal, 36–240] pg/mL) levels indicated aldosterone-resistant PHA.

Ureteral stenting to resolve the bilateral hydronephrosis and ureteral stricture immediately reduced serum aldosterone and renin levels, normalized the blood pressure, and improved electrolyte abnormalities (Fig. 1). Presuming that decreasing urinary tubular pressure enhanced aldosterone response inside the tubular cells, obstructive nephropathy-associated TPHA was diagnosed.

**Fig. 1:**
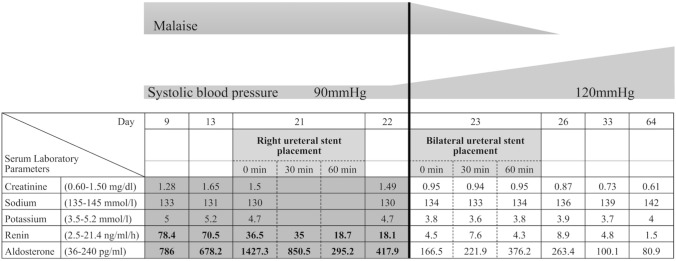
Renin and aldosterone concentrations and renal function rapidly improved after bilateral ureteral stent placement. Hypotension and fatigue were simultaneously mitigated

Renal biopsy (day 8) indicated secondary membranous nephropathy; prostate biopsy (day 100) revealed prostatic adenocarcinoma (Gleason score, 4 + 5); urine analysis for cytology (day 114) revealed urothelial carcinoma cells. The patient underwent orchiectomy for prostate cancer, and is currently undergoing treatment for ureteral cancer.

Transient aldosterone resistance can lead to TPHA. Here, metabolic acidosis with hyperkalemia suggested type 4 RTA. Obstructive uropathy may present type 4 RTA characteristics due to impaired H^+^ and K^+^ secretion in the collecting ducts [2]. In our case, despite high renin and aldosterone levels, hypotension suggested aldosterone resistance involvement in TPHA. Most TPHA cases are pediatric. An adult-onset idiopathic TPHA patient, with hydronephrosis and ureteral obstruction post-renal transplantation, reported improvement 4 months following ureteral reinsertion, suggesting hydronephrosis as the cause [3], as in our case. Aldosterone resistance in adult-onset TPHA may be caused by chronic inflammation and decreased filtration rate. Inflammatory conditions have high transforming growth factor levels, suppressing the aldosterone sensitivity of the collecting ducts [4]. Bilateral ureteral stenosis reduces the filtration rate; abnormal hydrostatic pressure on the principal cells of the collecting duct causes aldosterone resistance. Our patient showed immediate restoration to normotension and normalization of serum renin and aldosterone levels after stenting. Because of incomplete ureter occlusion, slight stenosis induced a subtle hydrostatic load. The extent of aldosterone resistance by hydrostatic loading requires further study.

TPHA, like hypoaldosteronism, presents with electrolyte abnormalities that are classified as type 4 RTA, but with distinct underlying mechanisms. Serum aldosterone levels should be monitored when treating hyperkalemia and metabolic acidosis, for determining the presence of aldosterone resistance. This may help diagnose hitherto hidden diseases such as ureteral tumors.

## Data Availability

The data underlying this article are available in the article and the figure captions.
